# Integrated Transcriptome and Metabolic Analyses Reveals Novel Insights into Free Amino Acid Metabolism in *Huangjinya* Tea Cultivar

**DOI:** 10.3389/fpls.2017.00291

**Published:** 2017-03-06

**Authors:** Qunfeng Zhang, Meiya Liu, Jianyun Ruan

**Affiliations:** ^1^Tea Research Institute, Chinese Academy of Agricultural SciencesHangzhou, China; ^2^Key Laboratory for Plant Biology and Resource Application of Tea, The Ministry of AgricultureHangzhou, China

**Keywords:** *Camellia sinensis*, chlorotic mutation, free amino acid, metabolism, nitrogen metabolism

## Abstract

The chlorotic tea variety *Huangjinya*, a natural mutant, contains enhanced levels of free amino acids in its leaves, which improves the drinking quality of its brewed tea. Consequently, this chlorotic mutant has a higher economic value than the non-chlorotic varieties. However, the molecular mechanisms behind the increased levels of free amino acids in this mutant are mostly unknown, as are the possible effects of this mutation on the overall metabolome and biosynthetic pathways in tea leaves. To gain further insight into the effects of chlorosis on the global metabolome and biosynthetic pathways in this mutant, *Huangjinya* plants were grown under normal and reduced sunlight, resulting in chlorotic and non-chlorotic leaves, respectively; their leaves were analyzed using transcriptomics as well as targeted and untargeted metabolomics. Approximately 5,000 genes (8.5% of the total analyzed) and ca. 300 metabolites (14.5% of the total detected) were significantly differentially regulated, thus indicating the occurrence of marked effects of light on the biosynthetic pathways in this mutant plant. Considering primary metabolism, including that of sugars, amino acids, and organic acids, significant changes were observed in the expression of genes involved in both nitrogen (N) and carbon metabolism. The suite of changes not only generated an increase in amino acids, including glutamic acid, glutamine, and theanine, but it also elevated the levels of free ammonium, citrate, and α-ketoglutarate, and lowered the levels of mono- and di-saccharides and of caffeine as compared with the non-chlorotic leaves. Taken together, our results suggest that the increased levels of amino acids in the chlorotic vs. non-chlorotic leaves are likely due to increased protein catabolism and/or decreased glycolysis and diminished biosynthesis of nitrogen-containing compounds other than amino acids, including chlorophyll, purines, nucleotides, and alkaloids.

## Introduction

The flavor and quality of tea are attributed to the presence of polyphenols, alkaloids, and amino acids. In particular, each free amino acid (or amide) has its own taste–as one of or a combination of sweet, salty, sour, bitter, and umami–which is why amides are recognized as the principal contributor to the mellow taste of brewed green tea (Zhang and Ruan, 2016). The concentrations of total free amino acids (including amides) and polyphenols in tea leaves can range from 1 to 5% and 20 to 40% (Zhang and Ruan, 2016), respectively. However, in premium green teas, a lower ratio of polyphenols to amino acids is required to balance the astringent and the mellow tastes. The most abundant free amino acids found in the tea plants are theanine (Thea), glutamine (Gln), glutamic acid (Glu), and arginine (Arg) (Harbowy et al., 1997). As a unique amino acid of green tea, theanine is a natural constituent, with many health benefits (Juneja et al., 1999). Nevertheless, the actual contents of amino acids in tea plants are highly influenced by plant variety and nutrition and by the local environment. Further, as the biosynthesis of amino acids requires nitrogen from the soil and carbon from the air, both nitrogen nutrition (Ruan et al., 2010) and photosynthesis (Zhang et al., 2014) play vital roles in the synthesis of amino acids in plants. However, too high a light intensity or temperature (or both) will not favor the accumulation of amino acids in tea plants (Zhang et al., 2014).

Chlorotic tea leaves have enhanced levels of free amino acids, which improve the quality of made tea, thus imparting to it a higher economic value relative to the non-chlorotic varieties (Feng and Barker, 1992; Ma et al., 2012). Moreover, the chlorotic mutant is considered to be a valuable resource in studies of how to improve the quality of brewed green tea, and of the metabolism of chlorophylls in tea leaves. Hence, increasing attention has been paid to chlorotic tea plant species; these are now accepted as the most popular cultivars for green tea, and as such they have been widely cultivated in China (Li et al., 2011; Ma et al., 2012; Xiong et al., 2013).

*Camellia sinensis* (L.) O. Kuntze cv. *Huangjinya*, one of the albino tea plant species, produces yellow-colored young shoots/leaves–the harvested parts for tea production–but green-colored mature leaves (i.e., from the previous year) under normal conditions of sunlight (Li et al., 2016). Interestingly, this mutant shows non-chlorotic traits for both its young shoots and mature leaves when grown under low-light conditions. Moreover, most of the chlorotic leaves that emerge under sunlight can revert to the green color once the light intensity is reduced, which enables *Huangjinya* to tolerate and survive under a wide range of light conditions. Otherwise, *Huangjinya* has a response to light that is very different from that of the normal tea species, i.e., under high-light exposure, a higher level of flavonoids is always produced by normal tea, but under high light the chlorotic leaves of *Huangjinya* contain a dramatically lower content of flavonoids than under reduced sunlight growing conditions. Changes in specific genes and in chemical components–total polyphenols, total amino acids, and pigments–in the *Huangjinya* plant were reported in previous studies (Li et al., 2016); however, little detailed information has been reported on nitrogen and amino acid metabolism and its regulation in the *Huangjinya* and also in other chlorotic tea plant.

The activation of nitrogen metabolism and the accumulation of amino acids in the albino mutants have frequently been attributed to extensive protein degradation (Harbowy et al., 1997; Motohashi et al., 2012; Feng et al., 2014; Satou et al., 2014). For example, in a chlorotic mutant, a drastic increase in the free amino acid content and the recycling of internal ammonium has been detected, along with protein degradation (Feng and Barker, 1992; Satou et al., 2014). Moreover, numerous studies are attempting to gain key insights into the metabolic networks of chlorotic mutant plants (Zhou et al., 2013; Satou et al., 2014; Wang et al., 2014; Li et al., 2016). Previous research has found that genes related to the tricarboxylic acid (TCA) cycle and the oxidative pentose phosphate pathway (OPPP) were highly expressed in the chlorotic mutants–the former for ketoglutarate synthesis and the latter for providing reducing power for nitrate assimilation (Emes and Neuhaus, 1997). Therefore, the accumulation of amino acids in chlorotic leaves may represent composite results of a global regulation of nitrogen metabolism as affected by chlorosis. However, the underlying molecular mechanisms that increase the free amino acid content in leaves of chlorotic tea mutants have not yet been elucidated.

In the present study, we used an “omics” research strategy, consisting of transcriptomic and metabolomics analyses, to reveal the effect of chlorosis on the global metabolome and on biosynthetic pathways in a chlorotic tea mutant. The objective was to reveal the mechanisms behind the increased levels of free amino acids in the chlorotic leaves. As a consequence, this research is also of value for developing strategies to improve the quality of tea leaves for commercial production and brewing.

## Materials and methods

### Plant material

The natural mutant of *Camellia sinensis* (L.) (cv. *Huangjinya*) was planted in pots at the Tea Research Institute, Chinese Academy of Agricultural Sciences (TRI, CAAS). In March 2014, 60 pots (Four plants per pot) of tea plants that had uniform young shoots (i.e., one bud and one leaf) were selected for this experiment: half of the pots were treated with “High-density Polyethylene Tape Two-Pin Net” (60% shading of sunlight) and the remaining 30 pots were exposed to full sunlight for 10 days (Figure S1). Randomly selected samples of the young shoots which had one bud with two leaves were taken, immediately frozen with liquid nitrogen, and then stored at −70°C in an ultra-refrigerator. The sampling was repeated six times for the non-chlorotic and chlorotic plants.

### Electron microscopy

Transmission electron microscopy (TEM) was used to observe the ultrastructure of the chlorotic leaf. Chlorotic and non-chlorotic Leaf samples (c. 1 mm^2^) were fixed with a 2.5% glutaraldehyde solution overnight at 4°C. Ultrathin sections were cut and stained; these were viewed under a transmission electron microscope (TEM, Joel JEM-1230) at an accelerating voltage of 80 kV, as described by Li et al. (2016).

### RNA isolation, cDNA library construction, illumina deep sequencing, and data processing

Four samples from two biological replicates of the chlorotic and non-chlorotic plant groups were used for the transcriptomic analysis. Total RNA was extracted using Trizol reagent (Invitrogen, USA) following the manufacturer's protocol. The RNA integrity was confirmed by a 2100 Bioanalyzer (Agilent Technologies). The RNA samples for the transcriptome analysis were prepared using an Illumina kit, following the manufacturer's recommendations. The fragments were purified via agarose gel electrophoresis and enriched by PCR amplification to create a cDNA library. Sequencing and data processing (including the statistical analysis and selection of differentially expressed genes) were all performed following the methods described by Wang et al. (2015). The transcriptome data for all the samples were deposited into the NCBI Sequence Read Archive (SRA) database under the accession number SRP072792.

### Metabolome analysis using GC × GC-TOF/MS

All 12 samples obtained–six biological replicates from each chlorotic and non-chlorotic plant group–were used for the metabolomics analysis. The tea leaves were extracted and derivatized as described by Lisec et al. (2006) and Liu et al. (2012). Each 1 μL aliquot of the derivatized sample was injected in the splitless mode into the GC × GC ToF-MS (Agilent GC 6890N gas chromatograph and Leco Pegasus HT high-speed ToF mass spectrometer), and analyzed following Liu et al. (2016).

Total ion chromatograms (TIC) were processed using the automated data processing software, ChromaTOF (v3.30, Leco Co., CA, USA). Metabolite identification from these selected variables was achieved via the NIST 05 Standard mass spectral databases (NIST, Gaithersburg, USA). The resulting data containing sample information, peak retention times, and peak intensities, were normalized to the area of the IS (afterward the IS peaks were removed), and then mean-centered.

Univariate statistics were carried out using one-way ANOVA in SPSS software (v.15.0, SPSS Inc., Chicago, USA). The unit variance was scaled for further statistical analysis using SIMCA-P software (v.13.0, Umetrics, Umea, Sweden). To obtain a general overview of the variance in metabolites, an unsupervised principal component analysis (PCA) was performed. To obtain information on the differences in the metabolite composition of the samples, a supervised orthogonal projection to latent structure discriminant analysis (OPLS-DA) was performed. The “variable importance in the projection” (VIP) values of all the data from the 7-fold cross-validated OPLS model were taken as the coefficients for the metabolite selection. For the purpose of group discrimination, only those variables that had a VIP > 1.0 and |p(corr)| > 0.65 were considered relevant. After the multivariate approaches were completed, the significance of each metabolite in the group discrimination was further evaluated by the Student's *t*-test (*P* < 0.05).

### Quantitative real-time PCR analysis

Total RNA was isolated using an RNAplant plus kit (Tiangen, China). Complementary DNA (cDNA) was synthesized using a PrimeScriptTM RT reagent Kit (TaKaRa). Quantitative real-time PCR (qRT-PCR) was performed on the Applied Biosystems 7,300 machine (Carlsbad, USA). The primer pairs used for the qRT-PCR are shown in Table S4 and *GAPDH* was used as the reference gene. For each target gene, triplicate reactions were performed. Relative transcript levels were calculated against that of the internal control *GAPDH* using the formula 2^−ΔΔCt^.

### Quantitative determination of amino acids, chlorophylls, carotenoids, and ammonium

Free amino acids, chlorophylls, and carotenoids in the young shoot/leaves samples were measured using an automatic amino-acid analyzer (Sykam S-433D, Germany) and by high-performance liquid chromatography with diode array detector (HPLC-DAD, Waters, 2695–2998), as previously reported by Liu et al. (2016). Ammonium extraction, purification, and quantification were performed according to the protocol described in Brautigam et al. (2007). The endogenous ammonium concentration was determined using an NH_4_Cl standard curve.

## Results

### Phenotype and ultrastructure of chlorotic and non-chlorotic leaves

**Figure 1:**
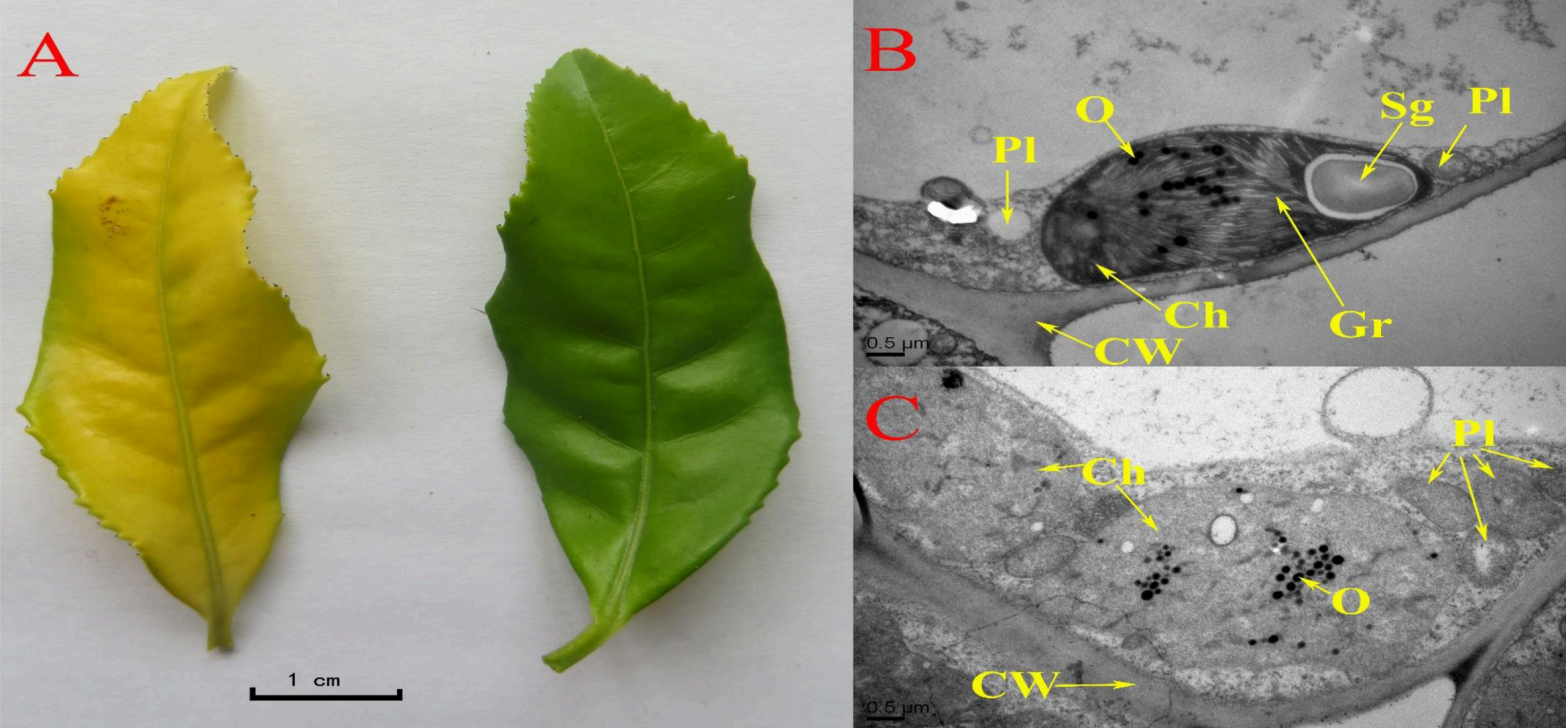
**Phenotypic and ultra-structural characterization of chlorotic and green leaves. (A)** Chlorotic leaves (left) and non-chlorotic leaves (right). **(B,C)** Ultrastructure of non-chlorotic leaves **(B)**, and chlorotic leaves **(C)**. Ch, chloroplast; CW, cell wall; Gr, grana; O, osmiophilic granules; pl, plastid; Sg, starch granule.

The leaves were chlorotic in the tea plants grown in full sunlight (sun, 800–2000 μmol m^−2^ s^−1^), while the leaves of plants shaded to 60% of the full sunlight intensity turned green (Figure 1A and Figure S2). The TEM analysis revealed apparent differences in the ultrastructure between the chlorotic and non-chlorotic leaves (Figures 1B,C). The chloroplasts of the chlorotic leaves were arrested at the proplastid stage, and they did not develop a clear sheet membrane, nor did they have grana structures but instead they had ubiquitous osmiophilic granules (Figure 1C). The membrane system and cellular compartmentalization of the chlorotic leaves were severely disrupted, and some chloroplasts showed signs of cavitation (Figure 1C). Interestingly, 10 days after chlorosis, the cells of the tea leaves reverted to a normal phenotype that had a fully developed chloroplast and a clear membrane structure (Figure 1B). Corresponding to the phenotypes, the chlorotic leaves contained significantly less chlorophyll (Table 1). The contents of chlorophyll-a and chlorophyll-b in the chlorotic leaves decreased by 96 and 77%, respectively, as compared with the non-chlorotic plants. The total content of carotenoids was decreased by 54% in the chlorotic leaves, whereas there were significant increases in their contents of zeaxanthin and carotene (Table 1).

**Table 1:** **The content (mg/100 g fresh weight) of chlorophylls and carotenoids in the young leaves of chlorotic (full sunlight) and non-chlorotic tea plants**.

	**Non-chlorotic plants**	**Chlorotic plants**
Chlorophyll total	57.81 ± 0.98	4.50 ± 0.08
Chlorophyll-a	45.61 ± 0.18	1.71 ± 0.05
Chlorophyll-b	12.20 ± 0.80	2.79 ± 0.04
Carotenoids	30.40 ± 0.08	13.87 ± 0.21
Neoxanthin	5.82 ± 0.06	0.39 ± 0.01
Lutein	18.02 ± 1.20	3.58 ± 0.04
Violaxanthin	1.22 ± 0.04	0.57 ± 0.02
α-Carotene	1.34 ± 0.09	1.90 ± 0.19
β -Carotene	2.86 ± 0.05	4.18 ± 0.26
Zeaxanthin	1.14 ± 0.06	3.25 ± 0.06

*Values shown are means ± SD (n = 3)*.

*All the listed metabolites showed a significant difference between the within-row means at P < 0.05*.

### Overview of the transcriptomic and metabolomic analyses

The transcriptome showed RNA-Seq datasets of robust quality and reliable results for the transcriptome assembly (Table S1). In total, 5051 (8.5% of the total analyzed) differentially-expressed genes (DEGs) were selected from the chlorotic and non-chlorotic leaves for bioinformatics analysis. The functional classification of these DEGs using Gene Ontology (GO) suggested that in the chlorotic leaves the expression levels of the growth-related genes were down-regulated, whereas the transcription levels of genes related to plant defense were up-regulated (Table S2). The detailed GO terms revealed that the basic physiological metabolism in the chlorotic tea mutant was inhibited through a resetting of the cellular framework, thus altering the composition, structure, and function of the plasma membrane, and the synthesis of key photo-protection molecules, such as flavonoids, carotenoids, and vitamins (Table S2). Pathway analysis based on the Kyoto Encyclopedia of Genes and Genomes (KEGG) revealed 34 metabolic pathways related to plant defense responses, and to carbon and nitrogen metabolism, all of which differed significantly between chlorotic and non-chlorotic leaves (Table 2). These pathways included biosynthesis of amino acids, purine metabolism, and starch and sucrose metabolism. Phenylalanine metabolism and the phenylpropanoid biosynthetic pathway, which are closely related to flavonoid metabolism, were also significantly altered in the chlorotic leaves.

**Table 2:** **Kyoto Encyclopedia of Genes and Genomes (KEGG) classification of significantly (***P*** < 0.01, FDR < 0.01) enriched pathways, following transcriptomic analysis of differentially expressed genes**.

**ID^a^**	**Term**	**Down^b^**	**Up^c^**
ko00970	Aminoacyl-tRNA biosynthesis	1	0
ko00906	Carotenoid biosynthesis	4	2
ko04713	Circadian entrainment	0	3
ko00073	Cutin	1	3
ko00904	Diterpenoid biosynthesis	3	1
ko01212	Fatty acid metabolism	0	3
ko00941	Flavonoid biosynthesis	5	5
ko00480	Glutathione metabolism	4	8
ko00591	Linoleic acid metabolism	0	2
ko00980	Metabolism of xenobiotics by cytochrome P450	4	5
ko00902	Monoterpenoid biosynthesis	0	2
ko03015	mRNA surveillance pathway	3	4
ko00360	Phenylalanine metabolism	3	9
ko00940	Phenylpropanoid biosynthesis	8	10
ko04744	Phototransduction	0	2
ko04075	Plant hormone signal transduction	10	6
ko04974	Protein processing	3	0
ko00620	Pyruvate metabolism	8	4
ko03010	Ribosome	4	7
ko03008	Ribosome biogenesis in eukaryotes	3	11
ko03018	RNA degradation	3	7
ko03013	RNA transport	1	7
ko00600	Sphingolipid metabolism	1	1
ko03040	Spliceosome	0	4
ko00500	Starch and sucrose metabolism	16	12
ko00900	Terpenoid backbone biosynthesis	7	2
ko00750	Vitamin B6 metabolism	1	2
ko00908	Zeatin biosynthesis	2	2

a *Pathway-map ID in the KEGG database (*http://www.genome.jp/kegg/*)*.

b,c *Numbers of genes down- or up-regulated in the chlorotic leaves (compared with leaves from the non-chlorotic leaves)*.

**Figure 2:**
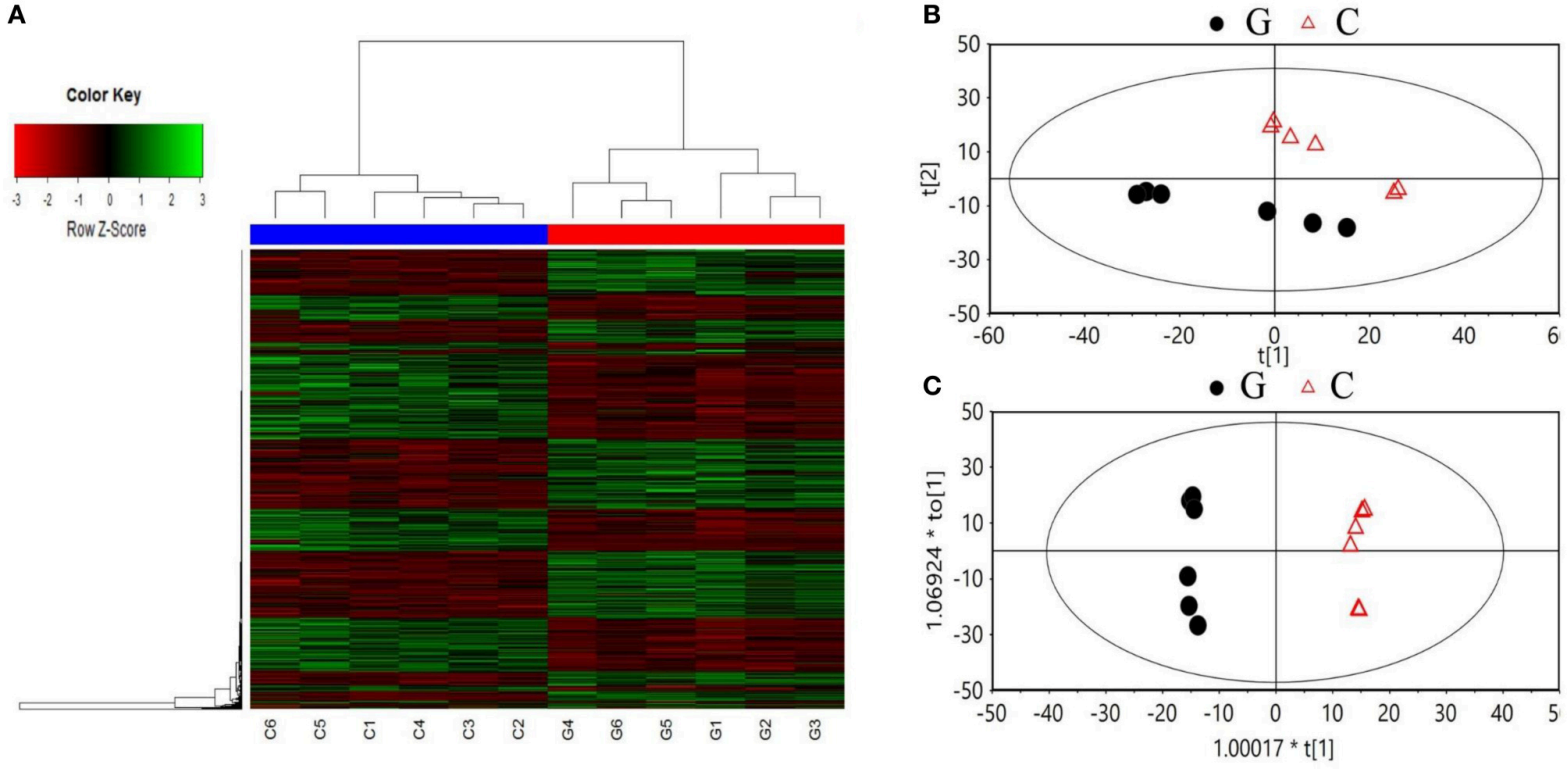
**Summary of the metabolic analysis**. Heat maps **(A)**, and the principal component analysis **(B)**, and projection to latent structure discriminant analysis **(C)** score plots of the metabolites analyzed by GC/GC-TOF MS in the non-chlorotic (G) and chlorotic (C) young tea leaves.

A total of 2,119 compounds were extracted from the raw data of the GC × GC-TOF/MS analysis. Both the PCA score plot and the heatmaps showed a clear separation between chlorotic and non-chlorotic leaves (Figure 2). The OPLS-DA modeling served to clarify those metabolites that were significantly affected by chlorosis. The summed variance explained by the components of the OPLS-DA model was 84.7% (Figure 2). The validation carried out via CV-ANOVA (ANOVA of the cross-validated residuals) confirmed that the models were not over-fitted (*P* = 3.28786e-005). Approximately 300 metabolites were significantly differentially regulated and 41 compounds related to the amino acid and carbon skeleton metabolism, the amino acids and derivatives, carbohydrates and organic acids and derivatives, have been identified (Table 3).

**Table 3:** **Significantly changed (VIP > 1 and |p(corr)| > 0.65 from partial least squares discriminant analysis) intracellular metabolites induced by chlorosis**.

**Compounds**	**VIP**	**p(corr)**	**Log2 (C/G)**
**AMINO ACIDS AND DERIVATIVES**			
L-Phenylalanine	1.59	0.83	0.26
L-Theanine	1.88	0.98	1.44
L-Glutamine	1.91	0.99	3.38
L-Lysine	1.91	0.99	2.58
L-Tyrosine	1.45	0.75	0.26
L-Glycine	1.46	0.75	1.19
L-Tryptophan	1.54	0.81	0.78
L-Alanine	1.90	0.98	4.85
L-Valine	1.87	0.96	1.03
L-Leucine	1.44	0.74	2.81
L-Serine	1.88	0.97	2.15
L-Aspartic acid	1.90	0.98	2.91
L-Methionine	1.53	−0.78	−1.49
L-Cysteine	1.67	0.86	1.74
L-Proline	2.21	0.94	2.64
L-Glutamic acid	1.24	0.78	1.44
**CARBOHYDRATES**			
Arabinose	1.51	−0.78	−0.66
L-Sorbose	1.24	−0.64	−1.91
D-Glucose	1.89	−0.97	−2.47
D-Galactose	1.59	−0.81	−0.74
D-Xylose	1.58	0.82	2.32
Pectin	1.86	0.96	1.98
D-Glucose	1.89	−0.97	−2.73
Fucose-1-P	1.24	0.94	0.55
Sucrose	1.58	−0.97	−1.56
Fructose 6-P	1.1	0.89	1.02
Fructose 2, 6-P2	1.42	−0.94	−1.32
Fructose	1.78	−0.94	−1.05
Mannose	1.67	−0.9	−2.34
Fucose	1.58	−0.99	−1.73
**ORGANIC ACIDS AND DERIVATIVES**			
cis-Aconitic acid	1.75	0.91	1.82
Tartaric acid	1.76	0.92	1.16
Oxalic acid	1.18	0.62	1.68
Citric acid	1.34	0.82	0.54
Digallate	1.08	0.67	0.40
Malic acid	1.37	−0.85	−0.57
Ascorbic acid	1.55	0.95	2.16
Phenylpyruvic acid	1.62	0.99	2.61
α-Ketoisovaleric acid	1.65	0.85	0.48
2-Oxovaleric acid	1.13	−0.58	−1.78
Vanillic acid	1.54	−0.94	−0.89

*VIP is the variable importance in the projection values from partial least squares discriminant analysis (PLS-DA); p(corr) is the correlation coefficient (ranging from −1.0 to 1.0) between the model and original data. The p(corr) values remain stable during iterative variable selection and are comparable between models; C/G is the ratio of the mean peak intensity in chlorotic (C, full sunlight) relative to non-chlorotic (G) tea plants*.

### Nitrogen and amino acid metabolism

The chlorotic leaves contained significantly more theanine and several other free amino acids than the non-chlorotic leaves (Tables 3, 4). Theanine, glutamic acid, glutamine, and aspartic acid were increased by 31.0, 39.8, 50.8, and 62.2%, respectively. The GC analytical results also showed that the chlorotic leaves had a higher content of amino acids (except for Met), derivatives of amino acids, and peptides and peptidomimetics than did the non-chlorotic leaves (Table 3). Among the increased amino acids, the most obvious were theanine, proline, aspartate, lysine, glutamine, and glutamate. Conversely, the biosynthesis of chlorophyll, nucleic acid, and several other nitrogen compounds was suppressed, and nitrogen utilization was reduced, in the chlorotic leaves. However, these phenomena were reversed in response to the shading treatment.

**Table 4:** **Content (mg/g fresh weight) of amino acids in young leaves of chlorotic (full sunlight) and non-chlorotic tea plants**.

	**Non-chlorotic plants**	**Chlorotic plants**
Alanine	0.28 ± 0.02a	0.55 ± 0.05b
Aspartic acid	0.90 ± 0.13a	1.46 ± 0.12b
Glutamine	0.61 ± 0.08a	0.92 ± 0.04b
Glutamic acid	2.41 ± 0.20a	3.37 ± 0.30b
Serine	0.30 ± 0.02a	0.41 ± 0.03b
Theanine	4.75 ± 0.30a	6.22 ± 0.20b
Threonine	0.07 ± 0.01a	0.09 ± 0.00a
Proline	0.06 ± 0.00a	0.07 ± 0.00a
Glycine	0.15 ± 0.01a	0.14 ± 0.02a
γ-Aminobutyric acid	0.05 ± 0.01a	0.05 ± 0.02a
${\text{NH}}_{4}^{+}$ (μmol/g fresh weight)	23.20 ± 2.07a	27.67 ± 1.33b

*Values shown are means ± SD (n = 3)*.

*Different letters within rows indicate a significant difference between the means at P < 0.05*.

**Figure 3:**
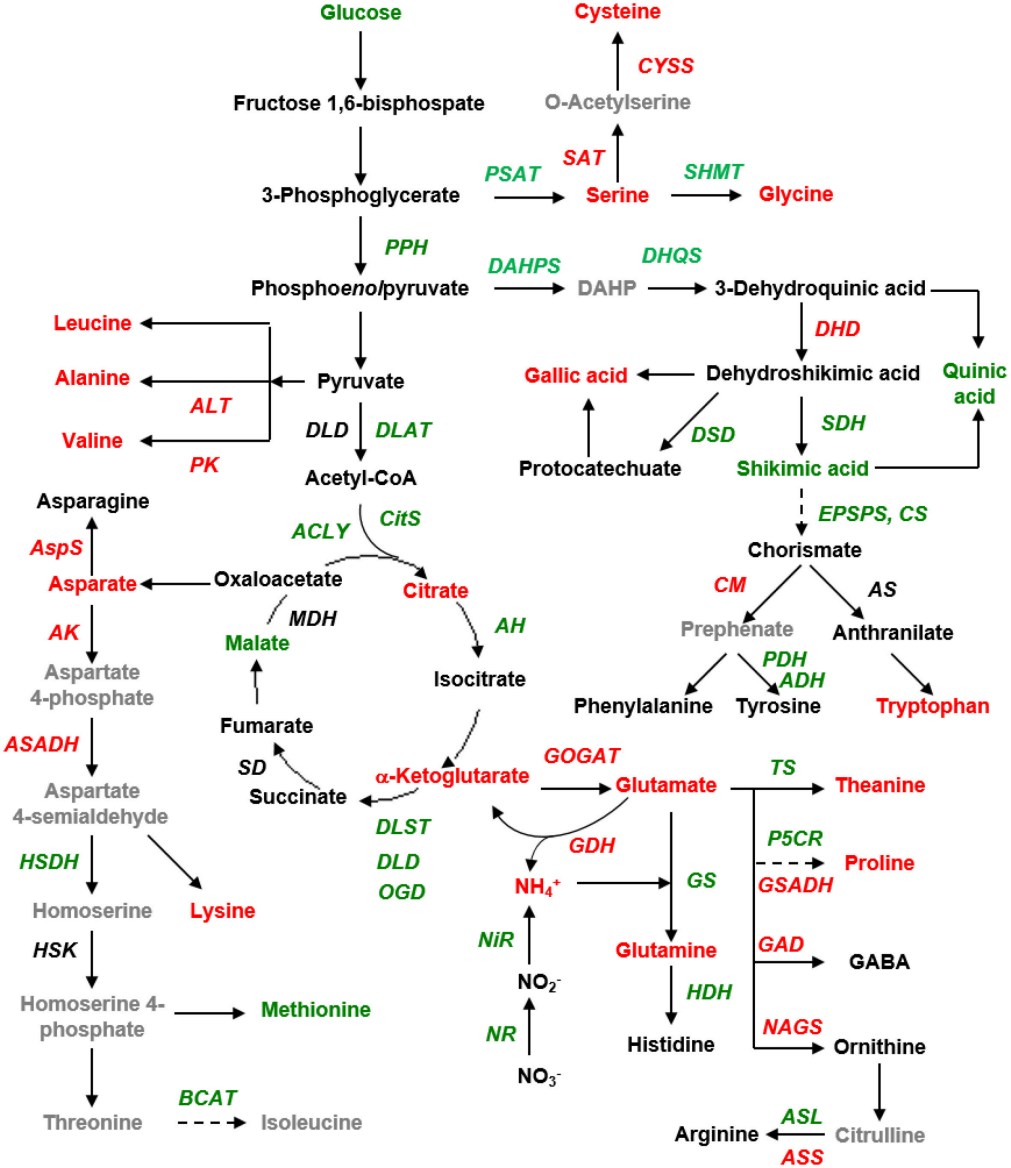
**Schematic presentation of the tricarboxylic acid cycle (TCA) pathway and amino acid biosynthesis, as affected by the chlorotic mutation**. Red and green fonts indicate the up- and down-regulated genes and metabolites in the chlorotic leaves as compared with those in the non-chlorotic leaves. The gray font indicates the genes and metabolites that were unidentified in this study. DAHP, 3-deoxy-D-arabino-heptulosonate-7-phosphate; PK, pyruvate kinase; PPH, phosphopyruvate hydratase; SAT, serine *O*-acetyltransferase; PSAT, Phosphoserine aminotransferase; CM, chorismate mutase; CS, chorismate synthase; DHD, 3-dehydroquinate dehydratase; DHQS, 3-dehydroquinate synthase; EPSPS, 3-phosphoshikimate 1-carboxyvinyltransferase; SDH, shikimate dehydrogenase; DSD, 3-dehydroshikimate dehydratase; ALT, alanine transaminase; AS, anthranilate synthase; Cyss, cysteine synthase; DAHPS, 3-deoxy-7-phosphoheptulonate synthase; SHMT, serine hydroxymethyltransferase; DLD, dihydrolipoyl dehydrogenase; DLAT, dihydrolipoyllysine-residue acetyltransferase; AspS, asparagine synthase; MDH, malic dehydrogenase (malate dehydrogenase); ACLY, ATP citrate synthase; CITS, citrate synthase; AH, aconitate hydratase; OGD, 2-oxoglutarate dehydrogenase (succinyl-transferring); DLST, dihydrolipoyllysine-residue succinyltransferase; SD, Succinate dehydrogenase; AK, aspartate kinase; ASADH, aspartate-semialdehyde dehydrogenase; HSDH, homoserine dehydrogenase; HSK, homoserine kinase; BCAT, branched-chain-amino-acid transaminase; NiR, ferredoxin-nitrite reductase; NR, nitrate reductase; GS, glutamate synthase; GOGAT, NAD(+)-dependent glutamate synthase; GDH, glutamate dehydrogenase; HDH, histidinol dehydrogenase; NAGS, *N*-acetylglutamate synthase; GSADH, glutamate-5-semialdehyde dehydrogenase; P5CR, pyrroline-5-carboxylate reductase; TS, theanine synthetase; ASS, argininosuccinate synthase; ASL, argininosuccinate lyase; GAD, glutamate decarboxylase. DHQD, 3-dehydroquinate dehydratase.

With regards to the assimilation of inorganic nitrogen into N-transport amino acids, we observed lower expression levels of the genes encoding ferredoxin-nitrite reductase (Fd-NiR), nitrate reductase (NR), glutamine synthetase (GS), and theanine synthase (TS) but higher expression levels of those encoding glutamate synthase (GOGAT), glutamate dehydrogenase (GDH), and asparagine synthase (AS) in the chlorotic as compared with the non-chlorotic leaves (Figure 3 and Table S3). The transcription of genes involved in asparaginase activity was inhibited in the chlorotic leaves, whereas genes involved in the activity of protein-disulfide reductase and *N*-acetyltransferase were up-regulated (Table S2). The expression levels of *GDH* and *GOGAT* were 2.2 and 1.3 times higher, respectively, in the chlorotic than in the non-chlorotic leaves.

In the pathway leading to the biosynthesis of proline, ornithine, arginine, histidine, and GABA, we observed higher expression levels in the chlorotic leaves than in the non-chlorotic leaves of the genes encoding *N*-acetylglutamate synthase (NAGS), glutamate-5-semialdehyde dehydrogenase (GSADH), glutamate decarboxylase (GAD), and argininosuccinate synthase (ASS), whereas the genes encoding pyrroline-5-carboxylate reductase (P5CR), histidinol dehydrogenase (HDH), and argininosuccinate lyase (ASL) showed a converse expression pattern (Figure 3).

The genes for alanine transaminase (ALT) and pyruvate kinase (PK), which are involved in the pathway leading to the synthesis of alanine and valine from pyruvate, were also up-regulated in the chlorotic leaves. In the biosynthetic pathway to the aspartate-derived amino acids, the expression levels of the genes encoding asparagine synthase (AS), aspartate kinase (AK), and aspartate-semialdehyde dehydrogenase (ASADH) were increased, while those of homoserine dehydrogenase (HSDH) and branched-chain-amino-acid transaminase (BCAT) were decreased.

In the aromatic amino acid biosynthetic pathway, we observed down-regulation in the chlorotic leaves of the genes encoding 3-deoxy-D-arabinoheptulosonate 7-phosphate synthase (DAHPS), 3-dehydroquinate synthase (DHQS), shikimate dehydrogenase (SDH), 5-enolpyruvylshikimate-3-phosphate (EPSPS), chorismate synthase (CS), prephenate dehydratase (PDH), and arogenate dehydrogenase (ADH, but conversely higher expression levels of the genes encoding 3-dehydroquinate dehydratase (DHD) and chorismate mutase (CM).

### Carbon skeleton metabolism

The abnormal development of the chloroplast and of the membrane structure in the chlorotic leaves inhibited photosynthesis, which impaired both glucose metabolism and carbon assimilation. Biological classification using GO suggested that in the “Cellular Component” classification, the expression of genes related to plastids and to membrane structure was strongly suppressed in the chlorotic leaves (Table S2). These genes were categorized as “chloroplast stroma,” “amyloplast,” and “integral component of membrane.” Photosynthesis-related genes were specifically suppressed in the chlorotic leaves. Under “carbon assimilation and glucose metabolism” within the “Molecular Function” classification, GO terms such as “electron carrier activity,” “starch synthase activity,” and “sugar transmembrane transporter activity” were mainly suppressed, whereas the expression of genes encoding trehalose phosphatase and trehalose phosphate synthase (UDP-forming) was enhanced in the chlorotic leaves (Table S2).

Metabolite profiling based on the GC × GC-TOF/MS showed that, compared to the non-chlorotic leaves, the chlorotic leaves had lower levels of most carbohydrates and their conjugates, such as glucose, fructose, sucrose, sorbose, arabinose, D-galactose, D-mannose, fucose, and fructose 2, 6-bisphosphate (Table 3). By contrast, the contents of D-xylose, fructose 6-phosphate, and fucose 1-phosphate were all distinctly increased in the chlorotic mutant. Moreover, the expression of genes encoding Rubisco, hexokinase (HK), phosphoglycerate mutase (PGM), fructokinase (FK), glycogen phosphorylase (GP), and others involved in photosynthesis and carbohydrate metabolism was up-regulated in the chlorotic leaves (Table S3).

Regarding the TCA cycle, the chlorotic leaves showed an increase in their ketoglutarate and citric acid contents, but a decrease in their malic acid content, as compared with the non-chlorotic leaves (Figure 3). The expression levels of genes encoding dihydrolipoyllysine-residue acetyltransferase (DLAT), ATP citrate synthase (ACLY), citrate synthase (CitS), aconitate hydratase (AH), oxoglutarate dehydrogenase (OGD), dihydrolipoyllysine-residue succinyltransferase (DLST), and dihydrolipoyl dehydrogenase (DLD)–involved in glycolysis and the TCA cycle–were all down-regulated in the chlorotic leaves as compared with the non-chlorotic (green) leaves (Figure 3).

## Discussion

*Huangjinya* showed two stark phenotypes of chlorotic vs. non-chlorotic when grown under normal and reducedlight conditions, respectively. Moreover, particular genes and metabolites were significantly differentially regulated in the chlorotic vs. non-chlorotic leaves. However, the effect of shading on *Huangjinya* differs completely from its effect on a normal tea species (Ku et al., 2009; Lee et al., 2013; Zhang et al., 2014) and other plant (Ding et al., 2016). For example, in the case of *Longjing* (Zhang et al., 2014), the amino acid content decreased significantly under high-light conditions as compared to lower-light (shade) treatment. By contrast, the amino acid content in *Huangjinya* was increased under high-light conditions.

**Figure 4:**
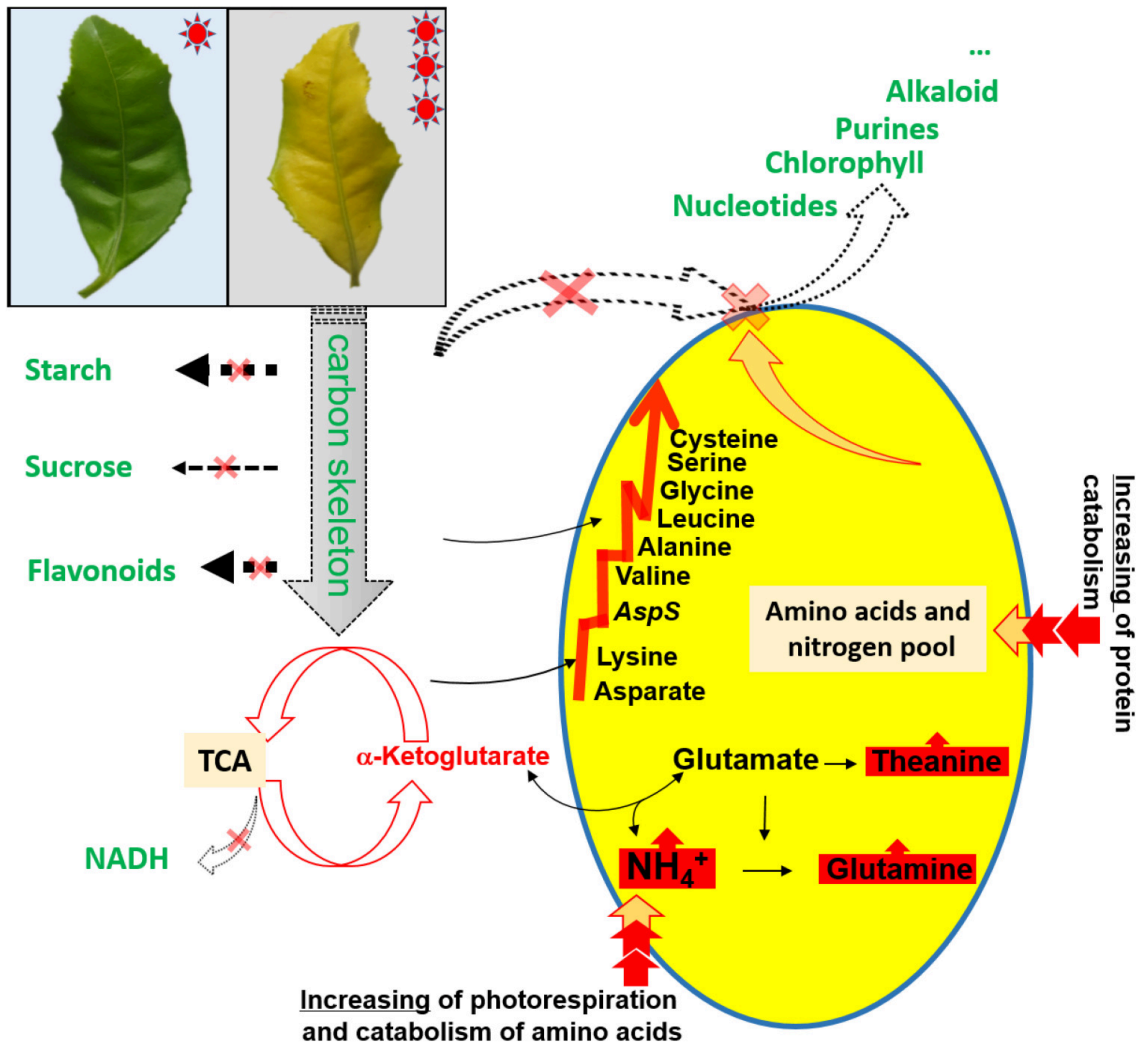
**Schematic presentation of amino acid metabolism as affected by the chlorotic mutation**.

Our experimental study demonstrated an abnormally developed chloroplast (Figure 1), impaired photosynthesis (Table 2 and Table S2), a reduced content of carbohydrates, reduced glucose metabolism, decreased flavonoid content, and an accumulation of amino acids (Tables 3, 4) in the chlorotic leaves. This indicates that the pathways related to C metabolism were all severely inhibited, which further disrupted the balance between carbon and nitrogen metabolism in the chlorotic leaves. A similar result was reported by Satou et al. (2014) for *Arabidopsis albino* (or pale green mutants), which show reduced photosynthetic rates and carbon metabolism but enhanced nitrogen metabolism. Returning to the present study, the results suggest that the chlorotic leaves were in a carbon-deficient condition: there were insufficient carbon skeletons present for the biosynthesis of amino acids (Figure 4). Moreover, the concentrations of *cis*-aconitic acid, citric acid, and ammonium all increased in the chlorotic leaves as compared with the non-chlorotic (green) leaves (Figure 3 and Table 4), thus indicating that the TCA cycle was activated even though carbon skeleton availability had been reduced significantly. These results are consistent with those of previous studies (Emes and Neuhaus, 1997; Satou et al., 2014). For example, Satou et al. (2014) suggested that the TCA cycle is activated to provide the GS/GOGAT cycle with 2-oxoglutarate for ammonium reassimilation.

However, we also observed in the chlorotic leaves a low level of expression for those genes involved in glycolysis and the TCA cycle, namely genes encoding PPH, DLAT, CITS, ACLY, AH, DLST, DLD, and OGD (Figure 3). This result may be attributable to the combined action of ammonium accumulation and a carbon supply shortage, or to feedback inhibition from the higher content of organic acids (i.e., TCA cycling products, which have a lower utilization efficiency in the etiolated mutant). A simplified metabolic network of the amine metabolites is shown in Figure 4, illustrating the disturbance of amino acid metabolism following exposure of the etiolated mutant to light.

In albino mutants, activation of nitrogen metabolism and accumulation of amino acids are often attributed to extensive protein degradation (Harbowy et al., 1997; Motohashi et al., 2012; Feng et al., 2014; Satou et al., 2014). In the present study, the transcriptomic analysis in relation to nitrogen metabolism showed clear evidence of protein degradation. For example, we observed a decrease in transcript levels related to amino acid biosynthesis (i.e., glutamate synthase) and an increase in the transcript levels of protein processing genes (Figure 3, Table 2). This indicated an obvious autophagy that was induced by damage to membrane structure and by the decomposition of macromolecules, which then increased the amino acid content in the chlorotic tea leaves. Moreover, we observed up-regulated expression of genes involved in the biosynthesis of Val, Ala, Asp, Asn, Lys, GABA, Orn, Arg, and Trp (Figure 3 and Table S3), which suggests an activated amino acid metabolism.

However, the accumulation of free amino acids may be due to low nitrogen consumption, as suggested by the decreased biosynthesis of chlorophylls. As been described by Scheible et al. (2004), the content of free amino acids will decreased as be consumed for biosynthesis of proteins. In particular, Glu is always used as the nitrogen source in the biosynthesis of nitrogen-containing compounds (Hammond-Kosack and Jones, 2000). A decrease in the biosynthesis of chlorophyll and other nitrogen-containing molecules should lead to the reduced consumption of precursor metabolites, mainly derived from glutamate–thus driving the accumulation of glutamate in the cells (Figure 4). Nonetheless, with the accumulation of nitrogen resources, and given the deficiency in carbon skeletons in the chlorotic tea leaves, the amino and nitrogen resources would need to be stored more efficiently: this is likely to be the reason why the amides glutamine, theanine and asparagine became the most abundant nitrogenous compounds in the albino leaves. The amide amino acids consist of a single carbon skeleton with two amino groups (Hammond-Kosack and Jones, 2000). Similarly, L-theanine, which only can be biosynthesized in root, probably accumulated because of continuous transportation from the roots and reduction of the consumption in the chlorotic leaves. In tea leaves, glutamine is converted into glutamate as a precursor to theanine biosynthesis, and the onward conversion of glutamate to theanine frequently occurs (Ruan et al., 2010). A high glutamate level accelerates the biosynthesis of theanine. Interestingly, the expression level of gene encoding theanine synthesis (TS) was down-regulated in the chlorotic leaves, likely via a negative feedback effect driven by its product. Similarly, the expression of *GS* was down-regulated, which may be explained by a negative feedback effect from the accumulation of glutamine and glutamate (Migge et al., 2000). Moreover, the decrease in flavonoid content (content of total catechins:16.7 and 17.6 mg/g in fresh weight of chlorotic and non-chlorotic leaves, respectively) should preserve carbon resources for amino acid biosynthesis, since the carbon in the ethylamine that is released during theanine catabolism is normally re-assimilated into flavonoids (Kito et al., 1968).

Our work revealed the higher accumulation of ammonium in the chlorotic leaves than in the non-chlorotic leaves (Table 4). This result agrees with previous reports that endogenous ammonium always increases in chloroplast-mutated plants, because of protein degradation (Harbowy et al., 1997; Satou et al., 2014). Endogenous ammonium is reportedly toxic in plant cells, and so it should be removed immediately (i.e., incorporated, Hammond-Kosack and Jones, 2000). The main enzymes responsible for endogenous ammonium metabolism are GOGAT and GDH. In the present study, the expression level of the *GOGAT* gene was up-regulated, which might be a mechanism to re-assimilate endogenous ammonium so as to mitigate any toxicity (Figure 3). The recycling of endogenous ammonium in chlorotic leaves should therefore promote nitrogen re-assimilation. The enzyme GDH catalyzes either the biosynthesis of glutamate, under a plentiful supply of endogenous ammonium, or conversely the catabolism of glutamate that generates ketoglutarate to fuel the citric acid cycle under carbon-limiting conditions (Melo-Oliveira et al., 1996). The expression level of the gene encoding GDH was increased in the chlorotic mutant (Figure 3). This result suggests that endogenous ammonium accumulates due to the decomposition of nitrogen-containing compounds in the chlorotic leaves, which also substantially promotes flux through the TCA. However, both a high level of endogenous ammonium and a carbon-pool shortage were detected, thus making it difficult to ascertain which factor played the dominant role.

Varying the levels of experimental shading is essential to understanding more fully the molecular mechanisms by which metabolism is altered in the chlorotic leaves. Pursuing this approach should clarify how the shade response to the influence of light intensity alters the biochemical profile in tea plants. Moreover, in the present work the reconstruction of the underlying metabolism was a non-trivial task, one that required both effective computational tools and a well-integrated knowledge-based system (Cho et al., 2008; Fukushima et al., 2014). Considering the complex relationships between the many different layers of biological information, which can only be captured by combining global measurements across these different levels, the integration of so-called “omics” data–transcriptomics, proteomics, and metabolomics–offers a promising approach to gaining a better understanding of various plant metabolisms.

## Conclusions

We studied the variation in gene expression patterns and metabolites between chlorotic and non-chlorotic leaves of the “*Huangjinya*” tea mutant plant using a combination of transcriptomic and metabolomic analyses. Our results reveal novel insights into nitrogen and amino acid metabolism. The supply of carbon skeletons is depleted in the chlorotic leaves; however, extensive protein degradation and decomposition of macromolecules increases the amino acid content. The accumulation of free amino acids is likely due to low nitrogen consumption and the highly efficient storage of nitrogen resources. Feedback regulation by ammonium, accumulated through photorespiration and protein decomposition in chlorotic leaves, may also promote TCA flux as well as the activation of nitrogen metabolism.

## Author contributions

QZ and ML gathered samples, participated in the study design, performed data analysis, interpreted the results and drafted the manuscript. JR conceived of the study, provided funding, and gave guidance on experimental design. All authors read and approved the final manuscript.

## Funding

This research was financially supported by the Ministry of Agriculture of China, through its Earmarked Fund for the China Agriculture Research System (CARS 23), and by the Chinese Academy of Agricultural Sciences through an Innovation Project for Agricultural Sciences and Technology (CAAS-ASTIP-2016-TRICAAS).

### Conflict of interest statement

The authors declare that the research was conducted in the absence of any commercial or financial relationships that could be construed as a potential conflict of interest.
