# Supplementary material for: Integrated Transcriptome and Metabolic Analyses Reveals Novel Insights into Free Amino Acid Metabolism in Huangjinya Tea Cultivar
Source: Front Plant Sci. 2017 Mar 6;8:291. doi: 10.3389/fpls.2017.00291 (PMC5337497; doi:10.3389/fpls.2017.00291)
Supplement: Supplementary file 1 [file Table1.DOCX]

**Table S1** Summary for the outcomes of de novo transcriptome assembly.

|  | **All (>=300bp)** | **>=500 bp** | **>=1000 bp** | **N50** | **Total Length** | **Max Length** | **Min Length** | **Average Length** |
| --- | --- | --- | --- | --- | --- | --- | --- | --- |
| Transcript | 261717 | 190074 | 124484 | 1876 | 335798953 | 24516 | 301 | 1283.06 |
| Unigene | 106499 | 55780 | 26857 | 1267 | 92494078 | 24516 | 301 | 868.5 |
