# Supplementary material for: Integrated Transcriptome and Metabolic Analyses Reveals Novel Insights into Free Amino Acid Metabolism in Huangjinya Tea Cultivar
Source: Front Plant Sci. 2017 Mar 6;8:291. doi: 10.3389/fpls.2017.00291 (PMC5337497; doi:10.3389/fpls.2017.00291)
Supplement: Supplementary file 2 [file Table2.DOCX]

**Table S2 Functional classification of differentially expressed genes using Gene Ontology**

|  |  | UP-regulated^a^ | | | | DOWN-regulated^b^ | | | |
| --- | --- | --- | --- | --- | --- | --- | --- | --- | --- |
| iD | Term | Genes^c^ | p | FDR | ES^d^ | genes | p | FDR | ES |
| ***Cellular component*** | | | | | | | | | |
| GO:0009507 | chloroplast | 102 | 0.04 | 0.11 | 1.17 | 96 | 0.02 | 0.06 | 1.23 |
| GO:0016021 | integral component of membrane | 90 | 1.00 | 1.00 | 0.70 | 144 | 0.00 | 0.02 | 1.24 |
| GO:0005576 | extracellular region | 49 | 0.00 | 0.00 | 1.70 | 35 | 0.03 | 0.09 | 1.35 |
| GO:0009570 | chloroplast stroma | 38 | 0.15 | 0.25 | 1.16 | 47 | 0.00 | 0.01 | 1.60 |
| GO:0005759 | mitochondrial matrix | 9 | 0.00 | 0.02 | 2.67 |  |  |  |  |
| GO:0005887 | integral component of plasma membrane | 4 | 0.11 | 0.21 | 1.57 | 5 | 0.03 | 0.08 | 2.18 |
| GO:0005815 | microtubule organizing center | 4 | 0.00 | 0.02 | 4.20 |  |  |  |  |
| GO:0000325 | plant-type vacuole | 3 | 0.26 | 0.37 | 1.16 | 5 | 0.03 | 0.09 | 2.16 |
| GO:0009534 | chloroplast thylakoid | 3 | 0.27 | 0.39 | 1.13 | 5 | 0.03 | 0.09 | 2.10 |
| GO:0005744 | mitochondrial inner membrane presequence translocase complex | 3 | 0.01 | 0.04 | 4.01 |  |  |  |  |
| GO:0042651 | thylakoid membrane | 3 | 0.01 | 0.04 | 3.76 |  |  |  |  |
| GO:0010282 | senescence-associated vacuole |  |  |  |  | 8 | 0.00 | 0.00 | 31.39 |
| GO:0010287 | plastoglobule |  |  |  |  | 6 | 0.03 | 0.09 | 1.99 |
| GO:0009501 | amyloplast |  |  |  |  | 5 | 0.00 | 0.01 | 4.80 |
| GO:0005758 | mitochondrial intermembrane space |  |  |  |  | 3 | 0.00 | 0.02 | 5.89 |
| GO:0000159 | protein phosphatase type 2A complex |  |  |  |  | 3 | 0.02 | 0.06 | 3.08 |
| ***Molecular function*** | | | | | | | | | |
| GO:0046872 | metal ion binding | 65 | 0.00 | 0.02 | 1.45 | 52 | 0.03 | 0.09 | 1.29 |
| GO:0005506 | iron ion binding | 44 | 0.00 | 0.00 | 1.78 | 43 | 0.00 | 0.00 | 1.94 |
| GO:0020037 | heme binding | 37 | 0.00 | 0.03 | 1.55 | 41 | 0.00 | 0.00 | 1.91 |
| GO:0009055 | electron carrier activity | 32 | 0.31 | 0.43 | 1.07 | 46 | 0.00 | 0.01 | 1.71 |
| GO:0005507 | copper ion binding | 29 | 0.00 | 0.00 | 2.33 | 10 | 0.56 | 0.66 | 0.89 |
| GO:0032440 | 2-alkenal reductase [NAD(P)] activity | 19 | 0.99 | 1.00 | 0.60 | 56 | 0.00 | 0.00 | 1.97 |
| GO:0008026 | ATP-dependent helicase activity | 16 | 0.01 | 0.04 | 1.83 | 2 | 0.99 | 1.00 | 0.25 |
| GO:0004601 | peroxidase activity | 14 | 0.00 | 0.01 | 2.52 | 9 | 0.03 | 0.09 | 1.80 |
| GO:0052689 | carboxylic ester hydrolase activity | 14 | 0.00 | 0.00 | 2.72 | 8 | 0.04 | 0.11 | 1.73 |
| GO:0003968 | RNA-directed RNA polymerase activity | 13 | 0.00 | 0.00 | 12.93 | 2 | 0.06 | 0.13 | 2.21 |
| GO:0004497 | monooxygenase activity | 10 | 0.04 | 0.11 | 1.65 | 15 | 0.00 | 0.00 | 2.75 |
| GO:0004805 | trehalose-phosphatase activity | 9 | 0.00 | 0.00 | 7.13 | 3 | 0.03 | 0.08 | 2.64 |
| GO:0047134 | protein-disulfide reductase activity | 9 | 0.00 | 0.01 | 3.33 | 3 | 0.23 | 0.33 | 1.23 |
| GO:0008568 | microtubule-severing ATPase activity | 9 | 0.00 | 0.03 | 2.51 | 1 | 0.84 | 0.89 | 0.31 |
| GO:0015238 | drug transmembrane transporter activity | 8 | 0.02 | 0.07 | 2.07 | 8 | 0.01 | 0.04 | 2.30 |
| GO:0008080 | N-acetyltransferase activity | 8 | 0.00 | 0.01 | 3.41 | 1 | 0.63 | 0.71 | 0.47 |
| GO:0031418 | L-ascorbic acid binding | 8 | 0.00 | 0.00 | 5.64 |  |  |  |  |
| GO:0004709 | MAP kinase kinase kinase activity | 7 | 0.97 | 0.99 | 0.50 | 26 | 0.00 | 0.01 | 2.06 |
| GO:0019825 | oxygen binding | 7 | 0.01 | 0.03 | 2.64 | 4 | 0.09 | 0.17 | 1.68 |
| GO:0004364 | glutathione transferase activity | 7 | 0.00 | 0.01 | 3.53 | 3 | 0.10 | 0.19 | 1.68 |
| GO:0043295 | glutathione binding | 7 | 0.00 | 0.00 | 12.35 | 2 | 0.01 | 0.06 | 3.92 |
| GO:0003825 | alpha,alpha-trehalose-phosphate synthase (UDP-forming) activity | 7 | 0.00 | 0.00 | 9.05 | 2 | 0.03 | 0.09 | 2.88 |
| GO:0005388 | calcium-transporting ATPase activity | 7 | 0.01 | 0.05 | 2.36 |  |  |  |  |
| GO:0047213 | anthocyanidin 3-O-glucosyltransferase activity | 6 | 0.08 | 0.17 | 1.59 | 10 | 0.00 | 0.01 | 2.96 |
| GO:0045431 | flavonol synthase activity | 6 | 0.00 | 0.01 | 4.08 | 4 | 0.01 | 0.05 | 3.03 |
| GO:0004462 | lactoylglutathione lyase activity | 6 | 0.00 | 0.01 | 3.95 | 2 | 0.16 | 0.25 | 1.46 |
| GO:0010294 | abscisic acid glucosyltransferase activity | 6 | 0.00 | 0.00 | 5.06 | 1 | 0.29 | 0.40 | 0.94 |
| GO:0052716 | hydroquinone:oxygen oxidoreductase activity | 6 | 0.00 | 0.02 | 3.53 | 1 | 0.45 | 0.56 | 0.65 |
| GO:0080043 | quercetin 3-O-glucosyltransferase activity | 6 | 0.00 | 0.02 | 3.33 | 1 | 0.48 | 0.59 | 0.62 |
| GO:0046556 | alpha-L-arabinofuranosidase activity | 6 | 0.00 | 0.00 | 8.62 |  |  |  |  |
| GO:0047085 | hydroxyphenylacetonitrile 2-monooxygenase activity | 5 | 0.00 | 0.00 | 5.71 | 5 | 0.00 | 0.00 | 6.35 |
| GO:0050592 | 4-hydroxyphenylacetaldehyde oxime monooxygenase activity | 5 | 0.00 | 0.00 | 5.71 | 5 | 0.00 | 0.00 | 6.35 |
| GO:0080044 | quercetin 7-O-glucosyltransferase activity | 5 | 0.00 | 0.02 | 3.73 | 1 | 0.34 | 0.45 | 0.83 |
| GO:0045486 | naringenin 3-dioxygenase activity | 5 | 0.00 | 0.00 | 12.93 |  |  |  |  |
| GO:0009044 | xylan 1,4-beta-xylosidase activity | 5 | 0.00 | 0.00 | 8.82 |  |  |  |  |
| GO:0050403 | trans-zeatin O-beta-D-glucosyltransferase activity | 4 | 0.00 | 0.02 | 4.43 | 7 | 0.00 | 0.00 | 8.63 |
| GO:0004807 | triose-phosphate isomerase activity | 4 | 0.00 | 0.00 | 9.13 | 1 | 0.06 | 0.13 | 2.54 |
| GO:0047893 | flavonol 3-O-glucosyltransferase activity | 4 | 0.00 | 0.00 | 8.17 | 1 | 0.07 | 0.15 | 2.27 |
| GO:0000175 | 3'-5'-exoribonuclease activity | 4 | 0.00 | 0.00 | 9.70 |  |  |  |  |
| GO:0032450 | maltose alpha-glucosidase activity | 4 | 0.00 | 0.01 | 6.21 |  |  |  |  |
| GO:0045548 | phenylalanine ammonia-lyase activity |  |  |  |  | 7 | 0.00 | 0.00 | 14.39 |
| GO:0009011 | starch synthase activity |  |  |  |  | 6 | 0.00 | 0.00 | 8.93 |
| GO:0005199 | structural constituent of cell wall |  |  |  |  | 5 | 0.00 | 0.00 | 6.17 |
| GO:0008253 | 5'-nucleotidase activity |  |  |  |  | 4 | 0.00 | 0.00 | 9.09 |
| GO:0004067 | asparaginase activity |  |  |  |  | 3 | 0.00 | 0.01 | 9.25 |
| GO:0016711 | flavonoid 3'-monooxygenase activity |  |  |  |  | 3 | 0.00 | 0.01 | 9.25 |
| GO:0043394 | proteoglycan binding |  |  |  |  | 3 | 0.00 | 0.00 | 11.77 |
| GO:0051119 | sugar transmembrane transporter activity |  |  |  |  | 3 | 0.00 | 0.00 | 10.79 |
| ***Biological process*** | | | | | | | | | |
| GO:0055114 | oxidation-reduction process | 138 | 0.01 | 0.04 | 1.21 | 168 | 0.00 | 0.00 | 1.64 |
| GO:0046686 | response to cadmium ion | 58 | 0.00 | 0.00 | 2.15 | 22 | 0.63 | 0.72 | 0.91 |
| GO:0006979 | response to oxidative stress | 34 | 0.00 | 0.00 | 2.37 | 16 | 0.15 | 0.24 | 1.24 |
| GO:0006612 | protein targeting to membrane | 29 | 0.00 | 0.00 | 2.44 | 14 | 0.12 | 0.21 | 1.31 |
| GO:0010363 | regulation of plant-type hypersensitive response | 29 | 0.00 | 0.00 | 2.44 | 14 | 0.12 | 0.21 | 1.31 |
| GO:0035556 | intracellular signal transduction | 27 | 0.00 | 0.01 | 2.00 | 16 | 0.11 | 0.19 | 1.32 |
| GO:0031348 | negative regulation of defense response | 27 | 0.00 | 0.00 | 2.89 | 11 | 0.14 | 0.23 | 1.31 |
| GO:0010200 | response to chitin | 26 | 0.00 | 0.00 | 2.29 | 7 | 0.80 | 0.87 | 0.69 |
| GO:0009867 | jasmonic acid mediated signaling pathway | 23 | 0.00 | 0.00 | 2.59 | 11 | 0.11 | 0.20 | 1.38 |
| GO:0006950 | response to stress | 21 | 0.00 | 0.01 | 1.82 | 13 | 0.16 | 0.25 | 1.25 |
| GO:0009738 | abscisic acid-activated signaling pathway | 20 | 0.00 | 0.00 | 2.35 | 10 | 0.15 | 0.24 | 1.31 |
| GO:0009862 | systemic acquired resistance, salicylic acid mediated signaling pathway | 19 | 0.00 | 0.00 | 2.57 | 14 | 0.00 | 0.02 | 2.11 |
| GO:0009693 | ethylene biosynthetic process | 17 | 0.00 | 0.00 | 4.23 | 1 | 0.88 | 0.92 | 0.28 |
| GO:0010310 | regulation of hydrogen peroxide metabolic process | 16 | 0.00 | 0.00 | 3.17 | 7 | 0.09 | 0.17 | 1.54 |
| GO:0009617 | response to bacterium | 16 | 0.00 | 0.00 | 4.60 | 4 | 0.20 | 0.31 | 1.28 |
| GO:0002679 | respiratory burst involved in defense response | 16 | 0.00 | 0.00 | 4.01 | 4 | 0.29 | 0.40 | 1.11 |
| GO:0009407 | toxin catabolic process | 15 | 0.00 | 0.01 | 2.47 | 5 | 0.47 | 0.57 | 0.91 |
| GO:0009615 | response to virus | 15 | 0.00 | 0.00 | 7.37 | 1 | 0.55 | 0.65 | 0.55 |
| GO:0019243 | methylglyoxal catabolic process to D-lactate | 14 | 0.00 | 0.01 | 2.62 | 9 | 0.02 | 0.08 | 1.88 |
| GO:0010286 | heat acclimation | 13 | 0.00 | 0.00 | 3.94 | 2 | 0.57 | 0.67 | 0.67 |
| GO:0010583 | response to cyclopentenone | 12 | 0.00 | 0.00 | 4.01 | 5 | 0.05 | 0.12 | 1.86 |
| GO:0051555 | flavonol biosynthetic process | 11 | 0.00 | 0.00 | 4.74 | 5 | 0.02 | 0.07 | 2.40 |
| GO:0005992 | trehalose biosynthetic process | 10 | 0.00 | 0.00 | 4.08 | 4 | 0.07 | 0.15 | 1.82 |
| GO:0060148 | positive regulation of posttranscriptional gene silencing | 10 | 0.00 | 0.00 | 35.3 |  |  |  |  |
| GO:0010025 | wax biosynthetic process | 10 | 0.00 | 0.00 | 7.46 |  |  |  |  |
| GO:0009266 | response to temperature stimulus | 8 | 0.01 | 0.01 | 2.33 | 2 | 0.60 | 0.69 | 0.65 |
| GO:0042335 | cuticle development | 8 | 0.00 | 0.00 | 4.37 | 1 | 0.49 | 0.59 | 0.61 |
| GO:0042991 | transcription factor import into nucleus | 8 | 0.00 | 0.00 | 6.34 |  |  |  |  |
| GO:0016441 | posttranscriptional gene silencing | 8 | 0.00 | 0.00 | 14.1 |  |  |  |  |
| GO:0000186 | activation of MAPKK activity | 7 | 0.97 | 0.99 | 0.50 | 26 | 0.00 | 0.01 | 2.05 |
| GO:0044262 | cellular carbohydrate metabolic process | 7 | 0.00 | 0.00 | 5.33 | 1 | 0.33 | 0.44 | 0.85 |
| GO:0009060 | aerobic respiration | 7 | 0.00 | 0.03 | 2.89 |  |  |  |  |
| GO:0019375 | galactolipid biosynthetic process | 6 | 0.18 | 0.28 | 1.31 | 16 | 0.00 | 0.00 | 3.88 |
| GO:0009835 | fruit ripening | 6 | 0.00 | 0.00 | 8.62 | 1 | 0.13 | 0.22 | 1.60 |
| GO:0045493 | xylan catabolic process | 6 | 0.00 | 0.00 | 6.85 |  |  |  |  |
| GO:0009612 | response to mechanical stimulus | 6 | 0.00 | 0.01 | 3.64 |  |  |  |  |
| GO:0016036 | cellular response to phosphate starvation | 4 | 0.75 | 0.82 | 0.64 | 14 | 0.00 | 0.01 | 2.50 |
| GO:0010411 | xyloglucan metabolic process | 4 | 0.00 | 0.00 | 11.1 | 1 | 0.04 | 0.10 | 3.08 |
| GO:0008643 | carbohydrate transport | 3 | 0.14 | 0.24 | 1.51 | 7 | 0.00 | 0.01 | 3.92 |
| GO:0019632 | shikimate metabolic process | 3 | 0.00 | 0.00 | 8.95 |  |  |  |  |
| GO:0009817 | defense response to fungus, incompatible interaction | 2 | 0.39 | 0.51 | 0.89 | 10 | 0.00 | 0.00 | 4.96 |
| GO:0009684 | indoleacetic acid biosynthetic process | 2 | 0.72 | 0.79 | 0.54 | 10 | 0.00 | 0.01 | 3.02 |
| GO:0009607 | response to biotic stimulus | 2 | 0.43 | 0.54 | 0.83 | 9 | 0.00 | 0.00 | 4.18 |
| GO:0006569 | tryptophan catabolic process | 2 | 0.57 | 0.67 | 0.68 | 9 | 0.00 | 0.01 | 3.41 |
| GO:0009687 | abscisic acid metabolic process | 2 | 0.01 | 0.04 | 5.17 | 4 | 0.00 | 0.00 | 11.51 |
| GO:0006857 | oligopeptide transport | 1 | 0.89 | 0.94 | 0.27 | 13 | 0.00 | 0.00 | 3.87 |
| GO:0006559 | L-phenylalanine catabolic process | 1 | 0.25 | 0.36 | 1.05 | 7 | 0.00 | 0.00 | 8.17 |
| GO:0009805 | coumarin biosynthetic process | 1 | 0.47 | 0.58 | 0.64 | 6 | 0.00 | 0.01 | 4.25 |
| GO:0009970 | cellular response to sulfate starvation | 1 | 0.22 | 0.33 | 1.14 | 5 | 0.00 | 0.00 | 6.35 |
| GO:0005978 | glycogen biosynthetic process | 1 | 0.19 | 0.30 | 1.25 | 4 | 0.00 | 0.01 | 5.57 |
| GO:0010023 | proanthocyanidin biosynthetic process | 1 | 0.08 | 0.16 | 2.16 | 3 | 0.00 | 0.01 | 7.19 |
| GO:0009800 | cinnamic acid biosynthetic process |  |  |  |  | 7 | 0.00 | 0.00 | 14.39 |
| GO:0006817 | phosphate ion transport |  |  |  |  | 5 | 0.00 | 0.01 | 5.02 |
| GO:0006090 | pyruvate metabolic process |  |  |  |  | 3 | 0.00 | 0.00 | 11.77 |

a and b, up / down -regulated in the chlorotic leaves compared to in shaded plants,

c, numbers of significantly changed gene,

d, ES: enrichment score.
