# Supplementary material for: Integrated Transcriptome and Metabolic Analyses Reveals Novel Insights into Free Amino Acid Metabolism in Huangjinya Tea Cultivar
Source: Front Plant Sci. 2017 Mar 6;8:291. doi: 10.3389/fpls.2017.00291 (PMC5337497; doi:10.3389/fpls.2017.00291)
Supplement: Supplementary file 4 [file Table4.DOCX]

Table S4 Primer sequences for quantitative RT-PCR

| **Genes** | **Forward Primer (5’ - 3’)** | **Reverse Primer (5’- 3’)** | |
| --- | --- | --- | --- |
| GDH | GGCTCAACTAATGACCTGGAAG | | ACTCACTCTTACTTAACTCACTTGG |
| GOGAT | GAGGCGTGATGTATGTAACTGATA | | GGAACCTGCTAAACCACAATAAAC |
| GS | ACCAACTACAGTACCAAGTCCAT | | CCATAAGCAGCAATGTGTTCCT |
