# Supplementary material for: Integrated Transcriptome and Metabolic Analyses Reveals Novel Insights into Free Amino Acid Metabolism in Huangjinya Tea Cultivar
Source: Front Plant Sci. 2017 Mar 6;8:291. doi: 10.3389/fpls.2017.00291 (PMC5337497; doi:10.3389/fpls.2017.00291)
Supplement: Supplementary file 5 [file Image1.PDF]

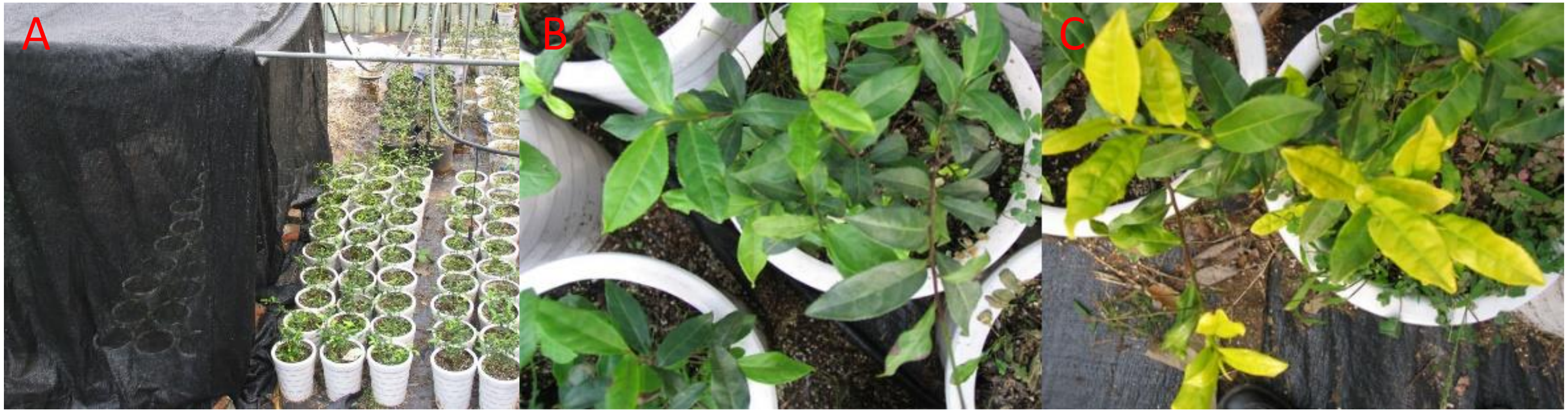

Fig. S1 "Huangjingya" tea plant treated with covering. A, the shading treatment; B, Green (shaded) plant; C, chlorotic plant.
