# Supplementary figures and images for: Integrated Transcriptome and Metabolic Analyses Reveals Novel Insights into Free Amino Acid Metabolism in Huangjinya Tea Cultivar
Source: Front Plant Sci. 2017 Mar 6;8:291. doi: 10.3389/fpls.2017.00291 (PMC5337497; doi:10.3389/fpls.2017.00291)

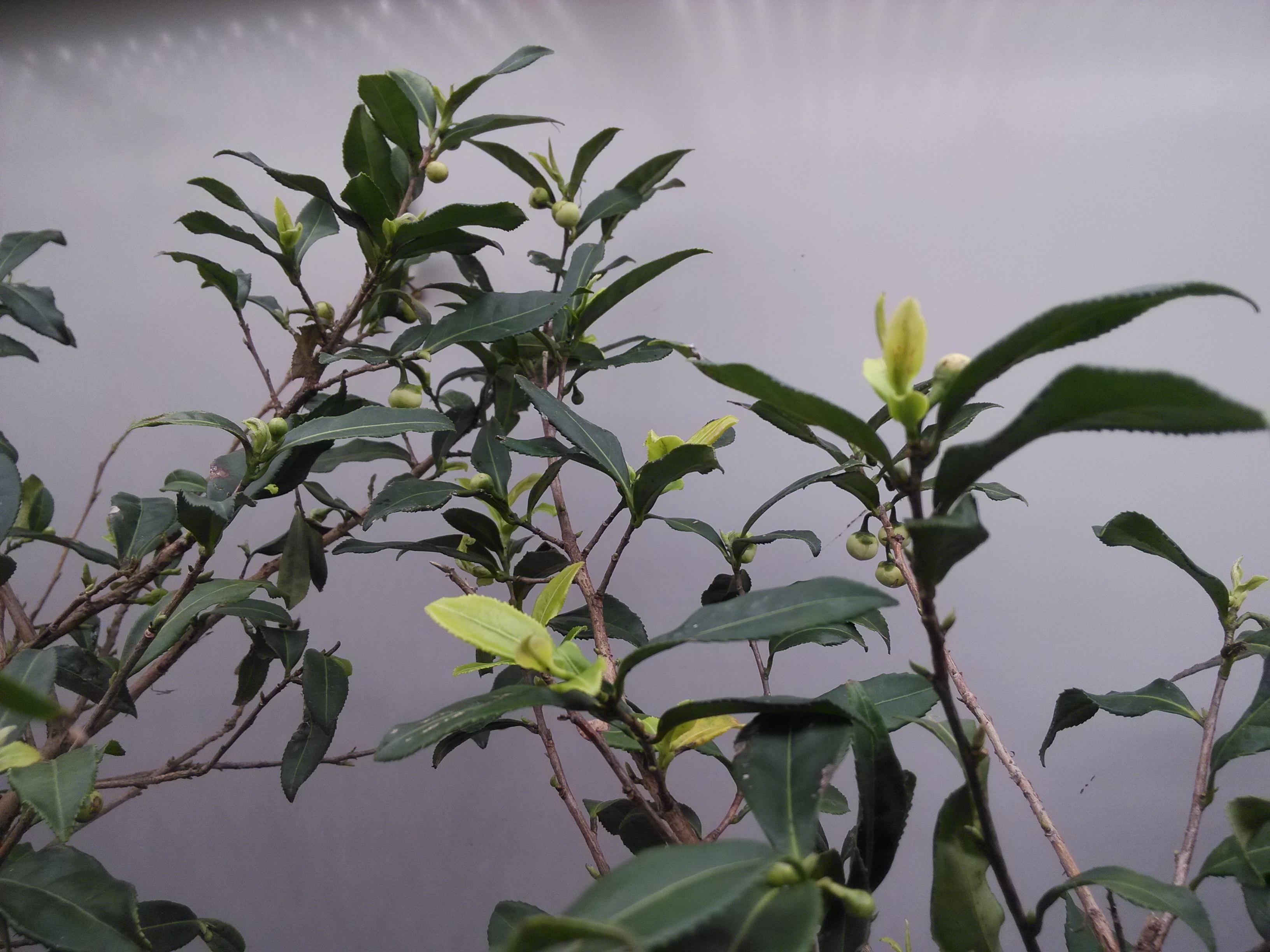

Supplement: Supplementary file 6 [file Image2.JPEG]
